# Phospholipid Mediated Activation of Calcium Dependent Protein Kinase 1 (*Ca*CDPK1) from Chickpea: A New Paradigm of Regulation

**DOI:** 10.1371/journal.pone.0051591

**Published:** 2012-12-20

**Authors:** Ajay Kumar Dixit, Chelliah Jayabaskaran

**Affiliations:** Department of Biochemistry, Indian Institute of Science, Bangalore, Karnataka, India; Griffith University, Australia

## Abstract

Phospholipids, the major structural components of membranes, can also have functions in regulating signaling pathways in plants under biotic and abiotic stress. The effects of adding phospholipids on the activity of stress-induced calcium dependent protein kinase (*Ca*CDPK1) from chickpea are reported here. Both autophosphorylation as well as phosphorylation of the added substrate were enhanced specifically by phosphatidylcholine and to a lesser extent by phosphatidic acid, but not by phosphatidylethanolamine. Diacylgylerol, the neutral lipid known to activate mammalian PKC, stimulated *Ca*CDPK1 but at higher concentrations. Increase in V_max_ of the enzyme activity by these phospholipids significantly decreased the K_m_ indicating that phospholipids enhance the affinity towards its substrate. In the absence of calcium, addition of phospholipids had no effect on the negligible activity of the enzyme. Intrinsic fluorescence intensity of the *Ca*CDPK1 protein was quenched on adding PA and PC. Higher binding affinity was found with PC (K_½_ = 114 nM) compared to PA (K_½_ = 335 nM). We also found that the concentration of PA increased in chickpea plants under salt stress. The stimulation by PA and PC suggests regulation of *Ca*CDPK1 by these phospholipids during stress response.

## Introduction

Plants have a novel family of protein kinases known as calcium dependent protein kinase (CDPK and which are biochemically distinct from other calcium dependent kinases as they require direct binding of calcium and which act independent of calmodulin [1]. Besides plants, CDPKs are also reported in *Plasmodium* [2], *Paramecium* [3], *Taxoplasma* [4]. But they are not present in the eukaryotic genome of yeast (*Saccharomyces cerevisiae*), nematodes [5], fruit flies (*Drosophila melanogaster*) [6] and humans [7]. CDPKs are widely distributed in plants and have diverse roles in gene expression [8], [9], metabolism [10]–[13], defense [14]–[16], development [17]–[19], ion transport [20]–[21] and salt/drought response [22]–[24].

CDPKs exist as monomeric serine/threonine protein kinases consisting of four domains: an amino-terminal variable domain, a kinase domain, an auto-inhibitory domain and a regulatory domain (CaM-LD - calmodulin-like domain). Many CDPKs are predicted to have myristoylation and palmitoylation sites at their N-terminal domain which are essential for membrane anchorage [25], [26].

In the absence of Ca^2+^, the auto- inhibitory domain acts as a pseudo-substrate which blocks the active site of the enzyme, thus keeping it in inactive state. However in presence of Ca^2+^, the enzyme undergoes conformational changes which remove the blocking and thus bringing the enzyme in active state [5].

The plasma membrane is a selective barrier between living cells and their environments and plays a key role in the perception and transmission of external information during stress condition. However, it can also serve as precursor for the generation of molecules like Inositol trisphosphate (IP3), Diacylgylcerol (DAG), Phosphatidylserine (PS), Phosphatidic acid (PA) etc. during stress exposure [27]. Phosphatidylcholine (PC) is the most abundant phospholipid in eukaryotic membranes and exclusively present in membranes. Increase in PC concentration was found during drought, osmotic stress and cold stress [28], [29] suggesting its possible increased turnover in response to stress.

PA is known to regulate activities of many kinases like MAPK [30], *At*PDK1 [31] or phosphatases like ABI1 [32]. Involvement of PA in signaling and healing wounds was indicated by its binding to wound-specific *Zm*CPK11 [33]. Addition of phosphatidylinositol (PI), lysophosphatidylcholine (LysoPC) and crude phospholipids stimulated activities of an Oat CDPK and *At*CPK1 [34], [35] where as activity of recombinant *Dc*CPK1 was stimulated by PA, PS, PI and phosphatidylethanolamine (PE) [36]. CPK11 from maize showed stimulated activities in presence of PA, PS and PI, but not in presence of PC, LysoPC, PE, diolein, cardiolipin [37]. All these studies indicate the possible role(s) of phospholipid in regulating activity of CDPKs.

Earlier we have reported isolation and characterization of CDPK1 gene from *Cicer arietinum* (designated as *Ca*CDPK1) [38]. The expression of *Ca*CDPK1 in leaves was enhanced in response to high salt stress and fungal infection suggesting its functional role in salt stress and fungal infection [39].

Since it is known that osmotic stress increased PA and PC concentration, we decided to look at the possible role(s) of these phospholipids in regulation of *Ca*CDPK1 activity. We have also measured the catalytic parameters in presence of these phospholipids. In addition we also found that salt stress caused increase in concentration of PA in chickpea plants.

## Materials and Methods

Calcium chloride, EGTA, magnesium chloride and SDS were purchased from Sigma Chemical Company, St. Louis, USA. All other chemicals and solvents used were from Qualigens Fine Chemicals, India. ^32^P-ATP and ^32^P-H_3_PO_4_ were from Board of Radiation and Isotope Technology, Hyderabad, India. P81-phosphocellulose, TLC plates and 3 MM sheets from Whatman Ltd., UK and phospholipids, PA (1,2-diacyl-sn-gylcero-3-P sodium salt), PC (1,2-diacyl-sn-gylcero-3-phosphocholine), PE (1,2-diacyl-sn-gylcero-3-phosphoethanolamine),W7 (N-(6-Aminohexyl)-5-chloro-1-naphthalenesulfonamide hydrochloride) and diacylgylcerol were purchased from Sigma, USA. Chickpea seeds KAK2 variety were procured from University of Agricultural Science, Bangalore.

Crude phospholipid was isolated from egg yolk according to Bligh and Dyer method [40]. After extraction, phospholipids were concentrated by rotary evaporator and re-dissolved in chloroform: methanol (2∶1v/v), and stored in −20°C. Before each experiment the appropriate amount of crude phospholipid was dried under vacuum, re-suspended in 50 mM of Tris-HCl pH 7.2 and sonicated for 10–15 min.

### Expression and Purification of *Ca*CDPK1

Over-expression and purification of *Ca*CDPK1 was done as described previously [41]. In brief, the *Ca*CDPK1 ORF was sub-cloned in pRSET A vector and was transformed in *Escherichia coli* BL21 strain. Cells were grown at 37°C with vigorous shaking until an absorbance of 0.6 at 600 nm was reached. After induction with 0.1 mM of isopropyl-3-D-thiogalactopyranoside, cells were grown further for 4 h at 25°C and then harvested by centrifugation.

Recombinant *Ca*CDPK1 protein was purified under native conditions by affinity chromatography using Ni-NTA. Cells were re-suspended in lysis buffer pH 8.0 (50 mM Tris-HCl, 300 mM NaCl, 10 mM imidazole, 10% (v/v) glycerol, 1% (v/v) Triton X-100 containing 1 mM phenylmethylsulfonyl fluoride). Resuspended cells were lysed by adding lysozyme (1 mg/ml) followed by incubation for 30 min and sonication for 5–7 min in an ice-bath. Suspension was then centrifuged at 12000× g for 20 min. The supernatant was incubated with Ni -NTA slurry and mixed gently for 1 h at 4°C. The slurry was packed as a column and washed several times with wash buffer pH 8.0 (50 mM Tris-HCl, 300 mM NaCl, and 50 mM imidazole). Bound proteins were eluted with elution buffer pH 8.0 (50 mM Tris-HCl, 300 mM NaCl, and 300 mM imidazole). The protein elution was monitored by checking the fractions on 12% (w/v) SDS-polyacrylamide gel. Fractions containing the protein were pooled and dialyzed against the storage buffer (50 mM Tris -HCl pH 7.2, 150 mM NaCl, 1 mM DTT, and 10% glycerol) with a minimum of 4 changes. Protein concentrations were estimated by Bradford dye-binding method with BSA as the standard [42].

### Protein kinase assay

Autophosphorylation and histone phosphorylation assays were carried out according to a previously reported protocol [41]. In brief, the assay mixture contained histone III-S (1 mg/ml), Ca^2+^/EGTA buffer (50 mM Tris-HCl, pH 7.2, 10 mM MgCl_2_ and 1 mM EGTA) and 50 ng of the purified recombinant *Ca*CDPK1 in presence or absence of 1.2 mM CaCl_2_. Unless mentioned, in all assays calcium is used as Ca^2+^/EGTA buffer. Autophosphorylation assays were done in same conditions as substrate phosphorylation, except 500 ng of *Ca*CDPK1 was used and histone III-S was omitted. γ^32^-P ATP stock was prepared by mixing the 1 mM cold ATP with ^32^P labeled ATP. Reactions were initiated by addition of γ^32^-P ATP (300 cpm/pmole) and incubated for 10 min at 37°C. After 10 min the reactions were terminated by spotting it on P81 phosphocellulose papers. P81 papers were immediately washed three times with 150 mM H_3_PO_4_, and once with acetone. Papers were dried and ^32^P incorporation was counted in a liquid scintillation counter (Beckman Counter). For autoradiography the reactions were stopped by addition of 1× SDS loading dye and were separated on a 12% (w/v) SDS-PAGE and subjected to autoradiography.

To determine initial velocities in presence of phospholipids, protein kinase assays were carried out as described above using histone III-S at concentrations ranging from 1 to 200 µM, for 10 min presence of 200 µM of PA or PC. K_m_ and V_max_ were calculated from Lineweaver–Burk (1/V Vs 1/S) plot.

### Handling of lipids

Appropriate amounts of phospholipids were dissolved in chloroform/methanol (9∶1 v/v) and dried under vacuum and lipids were re-suspended in 50 mM of Tris-HCl pH 7.2 and sonicated till turbidity of lipid samples attains a constant value (10–15 min). The solutions were kept at room temperature for 30 min and then used in *Ca*CDPK1 assay.

### *In vivo* -^32^P labeling and salt treatment of chickpea

Chickpea (*Cicer arietinum* L. cv. Kabuli) seeds were sterilized and grown on wet paper towel for 5 days in dark. Salt treatment experiments were done according Darwish et al [43] with minor modifications. Equally grown 5 days old seedlings were transferred to 2.5 mM MES buffer (pH 5.7) and 10 mM KCl. For phospholipids labeling, 5 µCi ^32^P-H_3_PO_4_ was added per tube and incubated for 15 min. Salt treatments were started by transferring the seedlings to 2.5 mM MES buffer containing 300 mM NaCl for 15 min. Reaction was stopped by freezing the seedling in liquid nitrogen. Seedlings were crushed and 400 µl of chloroform/methanol/HCl (50∶100∶1, (v∶v∶v)) was added to the mixture, followed by vigorous shaking for 10 min. Two phases were induced by addition of 400 µl CHCl_3_ and 200 µl of 0.9% (w/v) NaCl. The organic phase was collected and mixed with 400 µl chloroform/methanol/HCl (3∶48∶47, (v∶v∶v)). Repeated shaking, spinning and removing the upper phase yielded a purified organic phase. The organic phase was dried by vacuum centrifuge and re-suspended in minimal amount of CHCl_3_. The phospholipids were separated on TLC using solvent system chloroform∶acetone∶methanol∶acetic acid:H_2_O(10∶4∶3∶2∶1(v/v)). Labeled PA was identified by co-migrating standard PA. Radioactivity was visualized by autoradiography and quantified by phosphoimaging. The difference in the amount of PA formed was calculated by subtracting the radioactivity of treated cells by that of non-treated seedlings.

### Fluorescence studies

Fluorescence emission spectra were recorded at 25°C in a Perkin-Elmer luminescence spectrophotometer. Intrinsic spectrum of *Ca*CDPK1 protein was recorded (1 µM) in buffer containing 50 mM Tris-HCl (pH 7.2), 150 mM NaCl and 1 mM DTT using 280 nm as excitation wavelength (slit 5 nm) and 300–420 nm (slit 5 nm) as emission range. *Ca*CDPK1was titrated with increasing amount of PA or PC vesicles. Changes in fluorescence at 341 nm (F_0_- F_i_) was plotted against phospholipid vesicle concentrations where F_0_ is fluorescence intensity at zero concentration of phospholipid vesicles and F_i_ is fluorescence intensity at given concentration of phospholipid vesicles. K_1/2_ was calculated from this graph. Care was taken to avoid scattering or inner filter effect. K_1/2_ values were calculated as the concentration of phospholipid required for a half-maximal change in fluorescence.

## Results

### Activation of *Ca*CDPK1 by phospholipids

**Figure 1:**
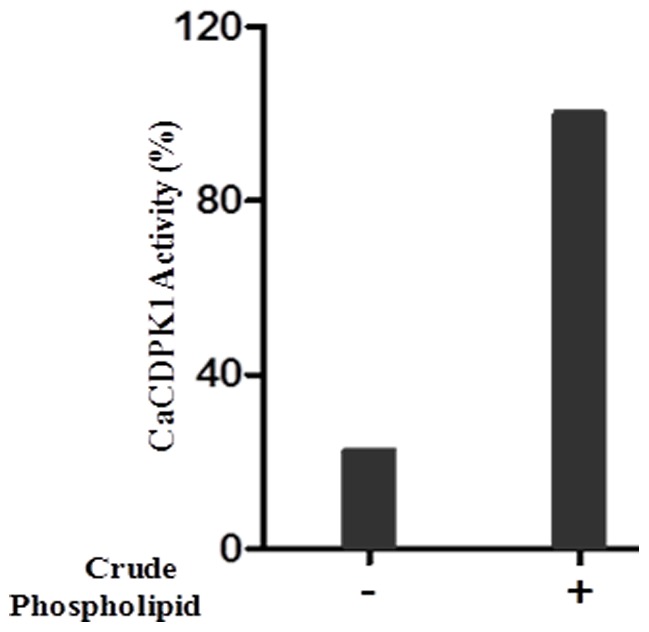
Effect of crude phospholipid on *Ca*CDPK1 activity. Kinase activity was performed in presence of crude phospholipid isolated from egg yolk. The reaction mixtures containing 50 ng of *Ca*CDPK1 in 50 mM Tris-HCl buffer (pH 7.2), 1.2 mM CaCl_2_, 1 mM EGTA, 10 mM MgCl_2_, 1 mg/ml histone III-S, and 50 µg of crude phospholipid were incubated at 37°C for 10 min, then spotted on P81 phosphocellulose papers and processed as described “Materials and methods” section. The activity is expressed as percentage with respect to the value in presence of crude phospholipid (100%). The result is representative of two independent experiments.

Using histone as exogenous substrate, kinase activity of *Ca*CDPK1 was measured in presence or absence of crude phospholipids. Crude phospholipid stimulated the kinase activity of the enzyme by 80 % indicating the requirement of phospholipid for maximum activity (Fig. 1).

**Figure 2:**
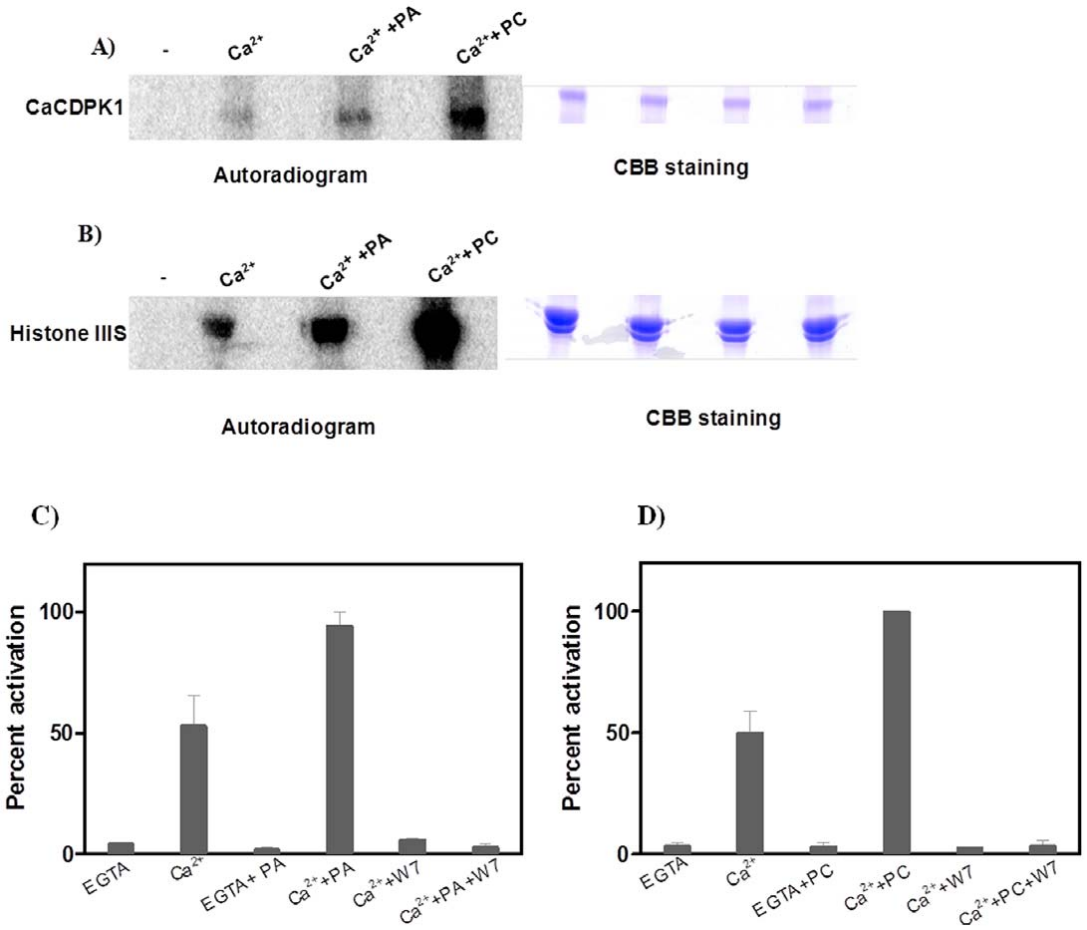
Autophosphorylation and histone phosphorylation activities of *Ca*CDPK1 in presence of added Ca^2+^, PA and PC. A) Autophosphorylation activity of *Ca*CDPK1 was measured in the presence of EGTA alone or Ca^2+^ alone, and Ca^2+^ with 100 µM PA or 100 µM PC, and 500 ng of kinase used per assay. B) Histone phosphorylation was measured in the presence of EGTA alone or Ca^2+^ alone, and Ca^2+^ with 100 µM PA or 100 µM PC. 50 ng of kinase and 1 mg/ml histone was used per assay. The reactions were stopped by adding 1× SDS loading buffer. The samples were run on 12% SDS-PAGE and subjected to autoradiogram. Parallel gels were run with *Ca*CDPK1 and histone, and stained with coomassie brilliant blue (CCB) for a loading control. C and D) Requirement of calcium for phospholipid dependent activation of *Ca*CDPK1. *Ca*CDPK1 activity was measure in EGTA alone, Ca^2+^ alone, EGTA and PA or PC, Ca^2+^ and PA or PC. To see the effect of W7, *Ca*CDPK1 activity was measured in presence of Ca^2+^ and W7 or Ca^2+^, W7 and PA or PC. 50 ng of *Ca*CDPK1, 1 mg/ml histone, 0.5 mM W7, 1 mM EGTA, 1.2 Ca^2+^ mM were used per assay. The reactions were stopped by spotting the reaction mixture on P81 phosphocellulose papers and were processed as described in “Materials and methods”.

PA stimulated autophoshorylation activity as well as histone phoshorylation activity (Fig. 2 A and B lane 3) of *Ca*CDPK1. In the presence PC autophosphorylation (Fig. 2A lane 4) and histone phosphorylation activities (Fig. 2 A and B lane 4) were stimulated to a higher degree than other phospholipids tested.

### Role of calcium and phospholipids in *Ca*CDPK1 activity

The activity of *Ca*CDPK1 was enhanced by PA and PC only in presence of calcium, as PA and PC failed to stimulate *Ca*CDPK1 activity in absence of calcium (Figure 2C and D). Adding N-(6-aminohexyl)-5-chloro-1-naphthalenesulphonamide (W7), a calmodulin antagonist, in assays, affected the calcium dependent activation as well as phospholipid dependent activation of *Ca*CDPK1 (Figure 2C and D). Together the results obtained indicated that *Ca*CDPK1 required Ca^2+^, for its enzyme activity, and the Ca^2+^-dependent activity was further enhanced by phospholipids.

**Figure 3:**
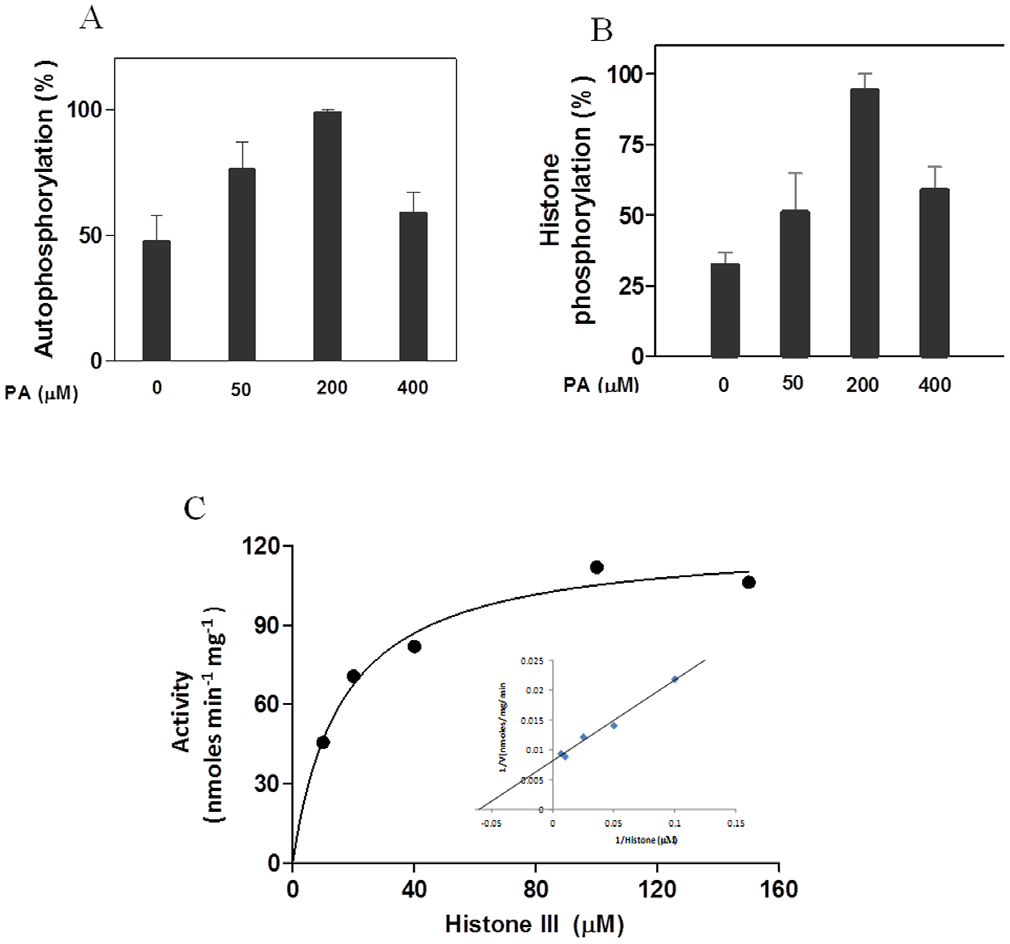
*Ca*CDPK1 activity in presence of PA. A) Autophosphorylation activity was measured in presence of increasing concentrations of PA. The reaction mixtures contained 500 ng of *Ca*CDPK1 in 50 mM Tris-HCl buffer (pH 7.2), 1.2 mM CaCl_2_, 1 mM EGTA, 10 mM MgCl_2_ and indicated amount of PA. Each value represents the mean ± SEM of triplicate measurements. The activity is expressed as percentage with respect to the highest ^32^P incorporation (100%) in presence of phospholipid (200 µM). B) Substrate phosphorylation activity was measured using histone IIIS as exogenous substrate in presence of increasing concentration of PA. Reaction mixture contained 50 ng of *Ca*CDPK1 in 50 mM Tris-HCl buffer (pH 7.2), 1.2 mM CaCl_2_, 1 mM EGTA, 10 mM MgCl_2_, 1 mg/ml histone III S and indicated amount of PA. Both sets of reaction mixtures were incubated for 10 min at 37°C and reactions were stopped by spotting the reaction mixture on P81 phosphocellulose papers. Papers were immediately processed as described in “Materials and methods”. Each value represents the mean ± SEM of triplicate measurements. The activity is expressed as percentage with respect to the highest ^32^P incorporation (100%) in presence of phospholipid (200 µM). C) Effects of PA on kinetics constant of recombinant *Ca*CDPK1. The plot of initial velocities versus histone concentrations for *Ca*CDPK1. Inset represents data plotted as the reciprocal of the velocities versus substrate concentrations, (1/(velocity) versus 1/(histone III-S)). Protein kinase assays were performed under standard conditions with 50 ng of pure recombinant *Ca*CDPK1 at different concentrations of histone III-S as the substrate and 200 µM of PA. Each value represents the mean of triplicate measurements and varies from the mean by not more than 15%.

PA stimulated autophosphorylation and substrate phosphorylation activities in a dose dependent manner (Fig. 3 A and B). Maximum activity was observed at a concentration 200 µM of PA. At this concentration, 58% stimulation in autophosphorylation activity and 62 % stimulation in histone phoshorylation activity were observed. At higher concentrations, however, PA seems to be inhibitory for the both activities of the enzyme.

Kinetics parameters were measured in presence of PA (200 µM) using histone as substrate (Fig. 3C). The values of V_max_ and Km were found to be 123 nmoles/min/mg protein and 16 µM in the presence of PA and 13.2 nmoles/min/mg proteins and 34.3 µM in the absence of any phospholipid [41], respectively (Table 1). Thus, addition of PA decreased the K_m_ value by 2 fold and increased the V_max_ value by 9-fold.

**Table 1:** Kinetic constants of *Ca*CDPK1 in the absence and the presence of PA and PC using histone IIIS as substrate.

	Vmax (nmoles min^−1^ mg^1^)	Km (µM)
No lipid	13.2	34.3
PA	123	16.0
PC	285	10.5

Effects of PA and PC on the catalytic parameters of *Ca*CDPK1 using histone III-S as substrate.

**Figure 4:**
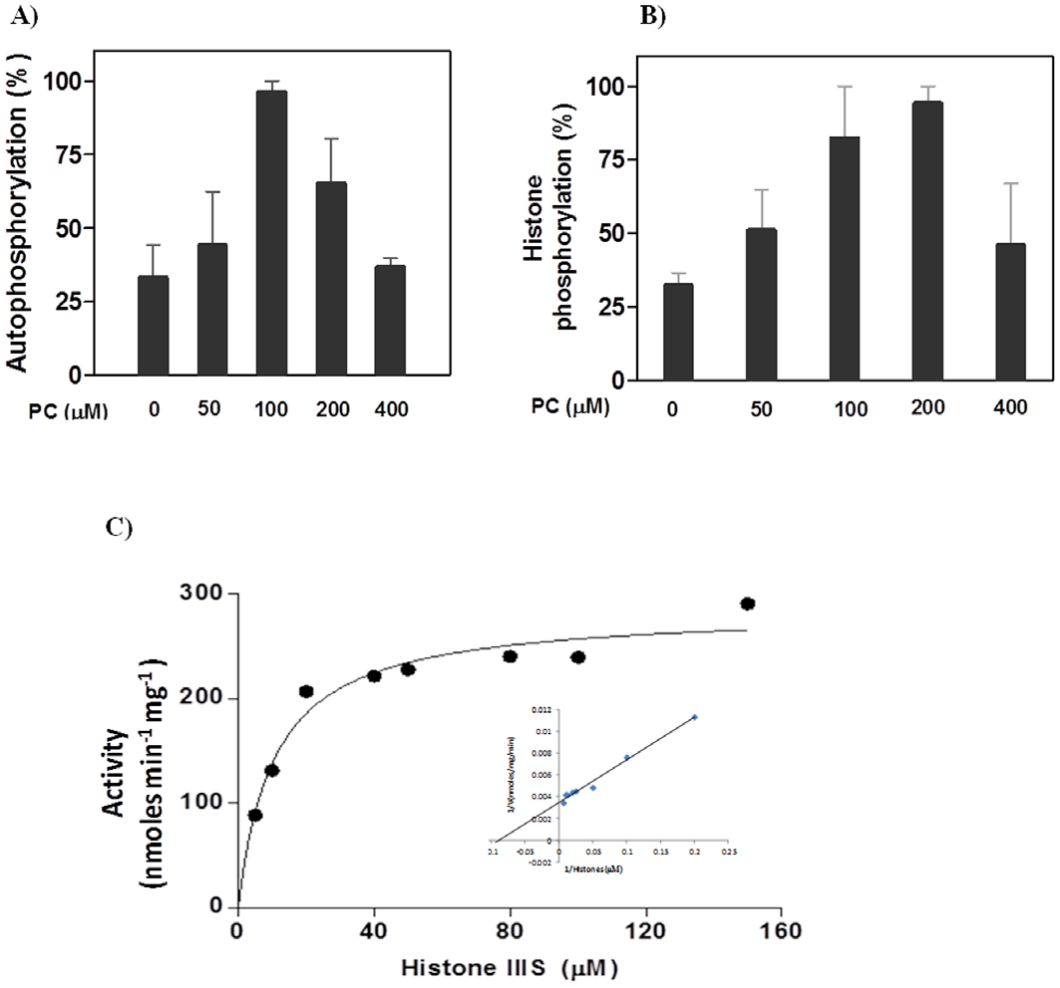
*Ca*CDPK1 activity in presence of added PC. A) Autophosphorylation activities were measured in presence of increasing concentrations of PC. Reaction mixtures contained 500 ng of *Ca*CDPK1 in 50 mM Tris-HCl buffer (pH 7.2), 1.2 mM CaCl_2_, 1 mM EGTA, 10 mM MgCl_2_ and indicated amount of PC. Each value represents the mean ± SEM of triplicate measurements. The activity is expressed as percentage with respect to the highest ^32^P incorporation (100%) in presence of phospholipid (100 µM). B) Histone phosphorylation activities were performed in presence of increasing concentration of PC. Reaction mixture containing 50 ng of *Ca*CDPK1 in 50 mM Tris-HCl buffer (pH 7.2), 1.2 mM CaCl_2_, 1 mM EGTA, 10 mM MgCl_2_, 1 mg/ml histone IIIS and indicated amount of PC. Both the sets of reaction mixtures were incubated for 10 mins at 37°C and reactions were stopped by spotting the reaction mixture on P81 phosphocellulose paper. The papers were processed immediately as described in “Materials and methods”. The activity is expressed as percentage with respect to the highest ^32^P incorporation (100%) in presence of phospholipid (200 µM). C) The plot of initial velocities versus histone IIIS concentrations. Protein kinase assays were performed under standard conditions with 50 ng of pure recombinant *Ca*CDPK1 at varying concentrations of histone III-S as the substrate and 200 µM of PC. Each value represents the mean of triplicate measurements. Inset represents the reciprocal plot of initial velocities versus substrate concentrations, (1/(velocity) versus 1/(histone III-S)).

Both activities of autophosphorylation and substrate phosphorylation were stimulated by PC in a dose dependent manner (Fig. 4 A and B). About 60% activation was observed at optimum concentration of PC. Catalytic parameters of the enzyme were calculated using histone as the substrate in presence of 200 µM of PC (Fig. 4C). The values of V_max_ and K_m_ of the enzyme were found to be 285 nmoles/min/mg protein and 10.5 µM (Table 1). In the presence of PC, 3.2-fold decrease in K_m_ and 21-fold increase in V_max_ were observed.

**Figure 5:**
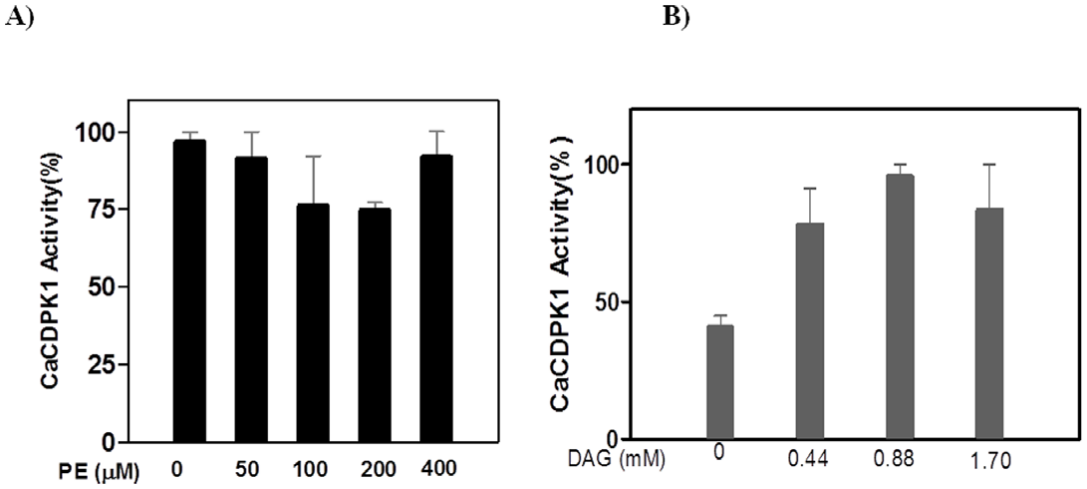
*Ca*CDPK1 activity in presence of added PE and diacylglycerol. A) Kinase activity was measured in presence of PE. Reaction mixtures contained 50 ng of *Ca*CDPK1 in 50 mM Tris-HCl buffer (pH 7.2), 1.2 mM CaCl_2_, 1 mM EGTA, 10 mM MgCl_2_, 1 mg/ml histone and indicated amount of PE. B) *Ca*CDPK1 activity in presence of high concentrations of diacylgylcerol. Kinase activity was measured in presence of diacylglycerol. The reaction mixture contained 50 ng of *Ca*CDPK1 in 50 mM Tris-HCl buffer (pH 7.2), 1.2 mM CaCl_2_, 1 mM EGTA, 10 mM MgCl_2_, 1 mg/ml histone and indicated amounts of DAG.

It is of interest to note that another important membrane phospholipid, PE, failed to stimulate *Ca*CDPK1 activity (Fig. 5 A). This indicated the specificity of PA and PC for activation of the enzyme.

### Lack of effect of diacylglycerol on *Ca*CDPK1 activity

DAG stimulated the activity of *Ca*CDPK1 only at high, unphysiological concentrations (Fig. 5B). At concentrations between 50–400 µM, comparable to those used for PC and PA, diacylgylcerol failed to stimulate the *Ca*CDPK1 activity (Fig. S1). The foregoing data demonstrate that PC is the most effective activator of this plant enzyme, *Ca*CDPK1, when compared to PA and DAG

### ^32^P incorporation into phosphatidic acid during salt stress

**Figure 6:**
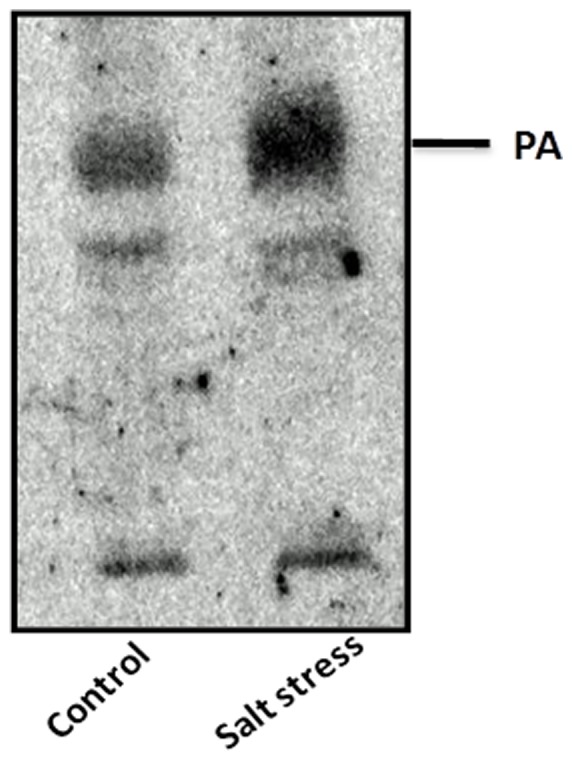
Effect of salt stress on ^32^ P-PA accumulations in chickpea plants. Chickpea seedlings were labeled for 15 min and then treated for 15 min with buffer (Control) or 300 mM NaCl. Phospholipids were extracted, separated on TLC. Labeled PA was identified by co-migration with standard PA using iodine staining. Radioactivity was visualized by autoradiography and quantified by phosphoimaging.

Increase in phosphatidic acid content in response to various environmental stress conditions is known in plants [43]. We investigated the response in 5-day old chickpea seedlings subjected to salt stress for 15 min by radio labeling PA with ^32^P-phosphate. Under the stress condition, incorporation of ^32^P into PA increased by about 2-fold (Fig. 6).

### Binding of phospholipid vesicles to *Ca*CDPK1

**Figure 7:**
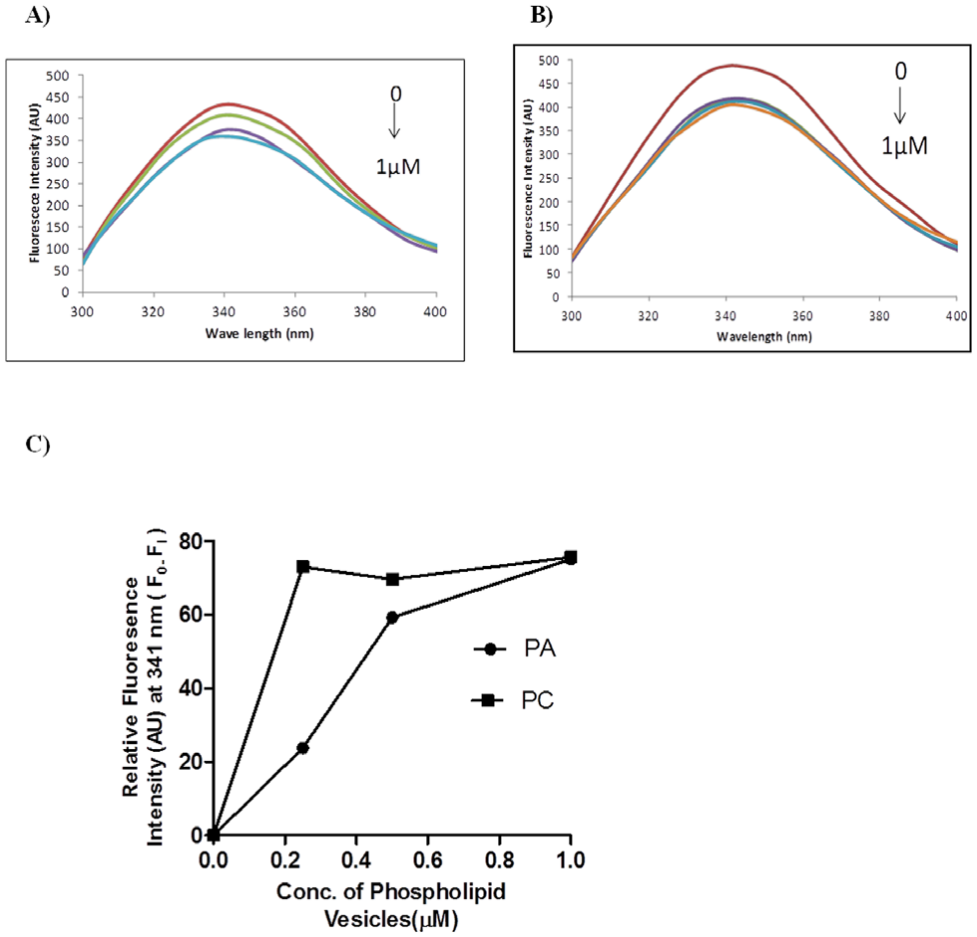
Binding of phospholipid vesicles to *Ca*CDPK1. Fluorescence emission spectra of *Ca*CDPK1 were recorded in the absence and the presence of increasing concentration of small unilamellar vesicles composed of PA (A) and PC (B). Samples were incubated in 50 mM Tris-HCl, pH 7.2,150 mM NaCl and 1 mM CaCl_2_, and titrated with increasing concentration of PA (red: *Ca*CDPK1 alone; green: 0.25 µM; purple: 0.5 µM; blue: 1 µM) and PC (red: *Ca*CDPK1 alone; blue: 0.25 µM; purple: 0.5 µM; orange: 1 µM). Samples were excited at 280 nm and the emissions from 300 to 400 nm were recorded. Excitation and emission slits were set at 5 nm. Care was taken to avoid inner filter effect or scattering. C) A re-plot of A and B showing change in fluorescence intensity at 341 nm as function of phospholipid vesicle concentration.

*Ca*CDPK1 exhibited an emission maximum of 341 nm showing the presence of tryptophan residues exposed to aqueous environment. Binding of PA and PC vesicles to *Ca*CDPK1 was monitored by recording fluorescence emission spectra in the presence of calcium with varying phospholipid concentrations. Changes in fluorescence intensity as the function of PA and PC concentration at a fixed *Ca*CDPK1 concentration are shown in Fig. 7A and B, respectively. Quenching of fluorescence emission of *Ca*CDPK1 on addition of phospholipid vesicles indicated re-localization of the tryptophan residues into a relatively more hydrophobic environment. Binding studies of phospholipid vesicles to *Ca*CDPK1 had to be carried out in a limited concentration range as lower concentrations did not induce significant change in fluorescence emission and higher concentrations caused scattering. Half maximal change in fluorescence intensity with PC (K_½_ = 114 nM) was lower than with PA (K_½_ = 335 nM) indicating more efficient binding of PC to *Ca*CDPK1, correlating with its higher activity (Fig. 7C).

## Discussion

Phosphatidic acid has emerged as a prominent signaling molecule during various biotic and abiotic stress conditions. Produced during stress either by PLD or by DAG/PLC-mediated pathway, PA regulates many proteins involved in stress physiology. The known targets of PA include Raf-1 [44], Opi1 [45], AtPDK1 [31], ABI1 [32], PEPC [46]. Actions of PA include increasing [31], [33], or decreasing [32] the enzymatic activities or changed localization of enzymes [44], [45].

Fluorescence emission spectroscopy showed quenching in fluorescence emission after binding to PA vesicles indicating that PA physically interacts with *Ca*CDPK1 and showed K_½_ of 335 nM. Several PA-binding proteins have been identified but there is no consensus sequence of the binding site. Different amino acid residues participate in PA binding in different proteins. Deletion of a KKR motif in the Opi1 transcription factor abolished PA binding to the protein. *Ca*CDPK1 protein also contains a KKR motif (Fig. S2) and its involvement in PA binding will require further studies on deletion of this motif.

Plants utilize one of their two major pathways for PA production, via phospholipase D (PLD) or phospholipase C/diacylgycerol kinase (PLC/DGK), depending on the nature of stress or signal [47]. Salt stress causes accumulation of PA by PLC/DGK pathway. Generation of PA by PLC/DGK, a fast reaction, occurs in min after imposing stress on the plant, and is usually monitored by the widely used method of incorporation of labeled ^32^P into diacylglycerol by DAK to produce PA. Our study confirmed increased incorporation of ^32^P in PA during salt stress indicating increased availability of PA.

PC is the most abundant phospholipid in eukaryotic membranes and exclusively present in membranes. Some studies showed that PC synthesis increased during salt stress, drought and cold stress [29] indicating its increased turnover during stress conditions, particularly the salt stress. PC is presently not considered signaling molecule. Till now not many CDPKs are known to be activated by PC. Pure PC did not significantly stimulate the kinase activity of *At*CPK1 [35] and *Zm*CDPK11 [33], and only small stimulation was found in case of Oat CDPK [34]. To best of our knowledge this is the first time we are reporting strong stimulation of CDPK by PC. Moreover, it was also reported that *At*PLAs are regulated by CDPK which enhanced *At*PLAs activities on phosphatidylcholine indicating involvement of CDPKs in PLA medicated signaling [48]. In view of selective and efficient activation of *Ca*CDPK1 by PC shown here, we propose that it may be involved in membrane-anchoring this protein in fully activated form. Activation of kinase and binding to *Ca*CDPK1 of PC was found to be stronger than PA hinting a specific role in regulating *Ca*CDPK1 activity. Indeed inter-conversion of PC and PA might afford the required modulation of the activity within a range of the already calcium-activated form of the enzyme and give an insight into lipid signaling during stress conditions.

## Conclusions

We demonstrate in the present study activation of *Ca*CDPK1 by PC and PA, but not by PE or diacylglycerol. Both phospholipids were able to bind to *Ca*CDPK1 and increased its V_max_ and affinity towards the exogenous substrate, histone.

## Supporting Information

Figure S1 *Ca*CDPK1 activity in presence of diacylgylcerol (50–400 µM). Kinase activity was measured in presence of diacylglycerol. The reaction mixture contained 50 ng of *Ca*CDPK1 in 50 mM Tris-HCl buffer (pH 7.2), 1.2 mM CaCl_2_, 1 mM EGTA, 10 mM MgCl_2_, 1 mg/ml histone and indicated amounts of DAG. Reactions were stopped by spotting the reaction mixtures on P81 phosphocellulose papers and were immediately processed as described in “Material and methods”.
(TIF)

Figure S2 Alignment of *Ca*CDPK1 an Opi1 amino acid sequence. KKR motif is shown in the box.
(TIF)
