# Supplementary figures and images for: Phospholipid Mediated Activation of Calcium Dependent Protein Kinase 1 (CaCDPK1) from Chickpea: A New Paradigm of Regulation
Source: PLoS One. 2012 Dec 20;7(12):e51591. doi: 10.1371/journal.pone.0051591 (PMC3527483; doi:10.1371/journal.pone.0051591)

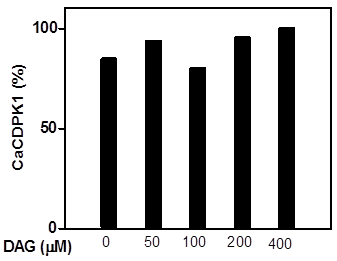

Supplement: Figure S1 — CaCDPK1 activity in presence of diacylgylcerol (50–400 µM). Kinase activity was measured in presence of diacylglycerol. The reaction mixture contained 50 ng of CaCDPK1 in 50 mM Tris-HCl buffer (pH 7.2), 1.2 mM CaCl2, 1 mM EGTA, 10 mM MgCl2, 1 mg/ml histone and indicated amounts of DAG. Reactions were stopped by spotting the reaction mixtures on P81 phosphocellulose papers and were immediately processed as described in “Material and methods”. (TIF) [file pone.0051591.s001.tif]

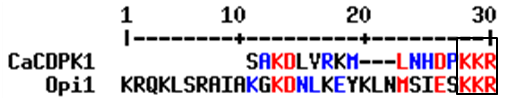

Supplement: Figure S2 — Alignment of CaCDPK1 an Opi1 amino acid sequence. KKR motif is shown in the box. (TIF) [file pone.0051591.s002.tif]
